# Supplementary material for: Foraging ecology of African wolves (Canis lupaster) and its implications for the conservation of Ethiopian wolves (Canis simensis)
Source: R Soc Open Sci. 2019 Sep 11;6(9):190772. doi: 10.1098/rsos.190772 (PMC6774988; doi:10.1098/rsos.190772)
Supplement: Supplementary Figure and tables [file rsos190772supp1.docx]

Supplementary materials


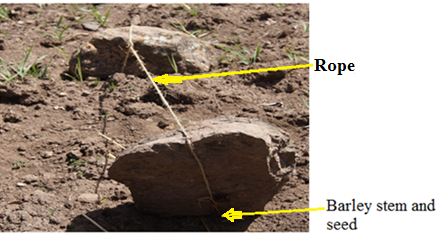


Figure S1. *Difit* amidst crops on farmland near GMCCA. The tip of the rope is tied to the barley (stem with seed). As the rodent feeds on the barley seed, the rope eventually breaks and the rock that is released kills the rodent.

Table S1. Diet of African wolves (total attempts and proportion that are successful recorded during tracking of 11 collared AWs at GMCCA and BSNP) based on focal follows (sus = successful, unsus = unsuccessful, sc = scavenging).

| N(n=2954 | | | dry | wet | suc | | unsuc | | sc |
| --- | --- | --- | --- | --- | --- | --- | --- | --- | --- |
| Food items | n | % | % | % | n | % | n | % |  |
| small rodents | 650 | 22.0 | 14.2 | 28.5 | 137 | 6.1 | 513 | 17 |  |
| insect | 561 | 19.0 | 24.0 | 14.8 | 561 | 24.8 |  |  |  |
| livestock carcass | 549 | 18.6 | 23.6 | 14.3 | 549 | 24.3 |  |  | 549 |
| rodent carcass | 359 | 12.2 | 2.9 | 20.0 | 359 | 15.9 |  |  | 359 |
| sheep | 192 | 6.5 | 8.9 | 4.5 | 34 | 1.5 | 158 | 5.4 |  |
| grass | 39 | 1.3 | 0.9 | 1.7 | 39 | 1.7 |  |  |  |
| mole rat | 23 | 0.8 | 0.4 | 1.1 | 2 | 0.1 | 21 | 0.7 |  |
| potato | 16 | 0.5 | 0.2 | 0.8 | 16 | 0.7 |  |  |  |
| wild bird | 6 | 0.2 | 0.4 | 0.1 | 6 | 0.3 |  |  |  |
| duiker | 5 | 0.2 | 0.3 | 0.1 | 5 | 0.2 |  |  |  |
| chicken | 4 | 0.1 | 0.2 | 0.1 | 4 | 0.2 |  |  |  |
| hare | 4 | 0.1 | 0.2 | 0.1 | 4 | 0.2 |  |  | 4 |
| unidentified | 547 | 18.5 | 23.9 | 14.0 | 547 | 24.2 |  |  |  |

Table S2. Twenty five farmlands assessed and rodents captured by *difit per hectare*.

| **Day** | **area (ha)** | | | **number of rodents** | | | **rodents captured ha^-1^** |
| --- | --- | --- | --- | --- | --- | --- | --- |
|  | **sum*** | **sd** | **mean** | **sum** | **sd** | **mean** |  |
| 1 | 2.8 | 0.9 | 0.4 | 55 | 19 | 11 | 19.6 |
| 2 | 2 | 0.7 | 0.4 | 39 | 4.1) | 7.8 | 19.5 |
| 3 | 2.5 | 0.3 | 0.5 | 38 | 3.2 | 7.6 | 15.2 |
| 4 | 2.8 | 0.3 | 0.6 | 33 | 4.7 | 6.6 | 11.8 |
| 5 | 1.8 | 0.1 | 0.4 | 32 | 2.3 | 6.4 | 17.8 |
| 6 | 2 | 0.2 | 0.4 | 32 | 3.2 | 6.4 | 16.0 |
| 7 | 4.5 | 0.3 | 0.6 | 107 | 8 | 13 | 23.8 |
| 8 | 1.8 | 0.1 | 0.4 | 36 | 3.1 | 7.2 | 20.0 |
| 9 | 2.3 | 0.3 | 0.5 | 63 | 4.8 | 12.6 | 27.4 |
| 10 | 5 | 0.3 | 0.5 | 88 | 4.6 | 9.8 | 17.6 |
| 11 | 3.5 | 0.2 | 0.4 | 68 | 4.5 | 10.2 | 19.4 |
| 12 | 2.5 | 0.3 | 0.5 | 60 | 3.7 | 7.8 | 24.0 |
| 13 | 2.8 | 0.3 | 0.6 | 41 | 3.1 | 8.2 | 14.6 |
| 14 | 3.7 | 0.2 | 0.4 | 99 | 6.2 | 12.2 | 26.8 |
| 15 | 1.5 | 0.1 | 0.4 | 45 | 8.6 | 11.3 | 30.0 |
| 16 | 2.2 | 0.2 | 0.3 | 75 | 7.9 | 13.2 | 34.1 |
| 17 | 1.8 | 0.1 | 0.4 | 43 | 4.7 | 8.6 | 23.9 |
| 18 | 1.8 | 0.1 | 0.4 | 32 | 3.2 | 6.4 | 17.8 |
| 19 | 2.3 | 0.3 | 0.5 | 78 | 8.8 | 15.6 | 33.9 |
| 20 | 2.3 | 0.3 | 0.5 | 84 | 6.5 | 16.8 | 36.5 |
| 21 | 2.8 | 0.3 | 0.6 | 54 | 7.9 | 10.8 | 19.3 |
| 22 | 2.5 | 0.3 | 0.5 | 63 | 6.5 | 12.6 | 25.2 |
| 23 | 1.5 | 0.1 | 0.4 | 31 | 4.1 | 6.2 | 20. 7 |
| 24 | 2.3 | 0.3 | 0.5 | 32 | 1.9 | 6.4 | 13.9 |
| 25 | 2.8 | 0.3 | 0.6 | 30 | 2.4 | 6 | 10.7 |
| 26 | 1.8 | 0.1 | 0.4 | 22 | 3.6 | 4.4 | 12.2 |
| 27 | 2 | 0.2 | 0.4 | 39 | 2.3 | 7.8 | 19.5 |
| 28 | 1.8 | 0.1 | 0.4 | 49 | 3.4 | 9.8 | 27.2 |
| 29 | 2.3 | 0.3 | 0.5 | 61 | 7.4 | 12.2 | 26.5 |
| 30 | 2.8 | 0.3 | 0.6 | 44 | 8.6 | 8.8 | 15.7 |
| 31 | 1.8 | 0.1 | 0.4 | 67 | 10.7 | 13.4 | 37.2 |
| 32 | 2 | 0.2 | 0.4 | 54 | 8.2 | 10.8 | 27.0 |
| 33 | 2 | 0.1 | 0.4 | 28 | 1.3 | 5.6 | 14.0 |
| 34 | 2 | 0.2 | 0.4 | 56 | 6.8 | 11.2 | 28.0 |
| 35 | 2 | 0.1 | 0.4 | 87 | 7.1 | 17.4 | 43.5 |
| 36 | 2.3 | 0.3 | 0.5 | 81 | 8.8 | 16.2 | 35.2 |
| 37 | 3 | 0.4 | 0.6 | 64 | 7.4 | 12.8 | 21.3 |
| 38 | 2 | 0.2 | 0.4 | 34 | 4.3 | 6.8 | 17.0 |
| 39 | 2 | 0.1 | 0.4 | 48 | 5.6 | 9.6 | 24.0 |
| 40 | 4.5 | 0.3 | 0.6 | 123 | 10.6 | 17.6 | 27.3 |
| 41 | 2.8 | 0.3 | 0.6 | 63 | 9.8 | 12.6 | 22.5 |
| 42 | 1.8 | 0.1 | 0.4 | 81 | 6.6 | 16.2 | 45.0 |
| 43 | 1.8 | 0.1 | 0.4 | 60 | 4.6 | 12 | 33.3 |
| 44 | 2.2 | 0.3 | 0.4 | 83 | 8.9 | 16.6 | 37.7 |
| 45 | 2.3 | 0.1 | 0.4 | 46 | 1.8 | 8.6 | 20.0 |
| 46 | 2.4 | 0.3 | 0.5 | 85 | 5.3 | 12.8 | 35.4 |
| 47 | 2.9 | 0.3 | 0.4 | 72 | 7.2 | 9.6 | 24.8 |
| 48 | 3.4 | 0.2 | 0.4 | 92 | 2.3 | 6.5 | 27.1 |
| 49 | 1.9 | 0.1 | 0.6 | 63 | 6.2 | 10.2 | 33.2 |
| 50 | 2.3 | 0.2 | 0.5 | 95 | 1.8 | 4.3 | 41.3 |
| 51 | 2.4 | 0.3 | 0.5 | 54 | 7 | 10.8 | 22.5 |
| sum | 124.3 | 12.2 | 23.6 | 3009 | 290.5 | 525.3 | 1258.1 |
| mean | 2.4 | 0.2 | 0.5 | 59 | 5.81 | 10.3 | 24.7 |
| sd | 0.7 | 0.1 | 0.1 | 23.4 | 3.2 | 3.5 | 8.5 |

Table S3 The differences of the frequency of food items between GMCCA and BSNP (food items as response variable, localities as fixed effect and individual collared animals as random samples).

|  | Value | Std.Error | DF | t-value | p-value |
| --- | --- | --- | --- | --- | --- |
| (Intercept) | 4.120203 | 0.114236 | 2941 | 36.0676 | 0 |
| Guassa | -0.08674 | 0.169494 | 9 | -0.51176 | 0.6211 |
